# HelicoBase: a *Helicobacter* genomic resource and analysis platform

**DOI:** 10.1186/1471-2164-15-600

**Published:** 2014-07-16

**Authors:** Siew Woh Choo, Mia Yang Ang, Hanieh Fouladi, Shi Yang Tan, Cheuk Chuen Siow, Naresh VR Mutha, Hamed Heydari, Wei Yee Wee, Jamuna Vadivelu, Mun Fai Loke, Vellaya Rehvathy, Guat Jah Wong

**Affiliations:** Genome Informatics Research Laboratory, High Impact Research (HIR) Building, University of Malaya, 50603 Kuala Lumpur, Malaysia; Department of Oral Biology and Biomedical Sciences, University of Malaya, 50603 Kuala Lumpur, Malaysia; Faculty of Medicine and Health Sciences, University Putra Malaysia, 43400 Serdang, Selangor Darul Ehsan Malaysia; Faculty of Computer Science and Information Technology, University of Malaya, 50603 Kuala Lumpur, Malaysia; Department of Medical Microbiology, Faculty of Medicine Building, University of Malaya, 50603 Kuala Lumpur, Malaysia

**Keywords:** HelicoBase, *Helicobacter*, Genomic resources, Pairwise genome comparison tool, Pathogenomics profiling tool, Comparative analysis

## Abstract

**Background:**

*Helicobacter* is a genus of Gram-negative bacteria, possessing a characteristic helical shape that has been associated with a wide spectrum of human diseases. Although much research has been done on *Helicobacter* and many genomes have been sequenced, currently there is no specialized *Helicobacter* genomic resource and analysis platform to facilitate analysis of these genomes. With the increasing number of *Helicobacter* genomes being sequenced, comparative genomic analysis on members of this species will provide further insights on their taxonomy, phylogeny, pathogenicity and other information that may contribute to better management of diseases caused by *Helicobacter* pathogens.

**Description:**

To facilitate the ongoing research on *Helicobacter*, a specialized central repository and analysis platform for the *Helicobacter* research community is needed to host the fast-growing amount of genomic data and facilitate the analysis of these data, particularly comparative analysis. Here we present HelicoBase, a user-friendly *Helicobacter* resource platform with diverse functionality for the analysis of *Helicobacter* genomic data for the *Helicobacter* research communities. HelicoBase hosts a total of 13 species and 166 genome sequences of *Helicobacter* spp. Genome annotations such as gene/protein sequences, protein function and sub-cellular localisation are also included. Our web implementation supports diverse query types and seamless searching of annotations using an AJAX-based real-time searching system. JBrowse is also incorporated to allow rapid and seamless browsing of *Helicobacter* genomes and annotations. Advanced bioinformatics analysis tools consisting of standard BLAST for similarity search, VFDB BLAST for sequence similarity search against the Virulence Factor Database (VFDB), Pairwise Genome Comparison (PGC) tool for comparative genomic analysis, and a newly designed Pathogenomics Profiling Tool (PathoProT) for comparative pathogenomic analysis are also included to facilitate the analysis of *Helicobacter* genomic data.

**Conclusions:**

HelicoBase offers access to a range of genomic resources as well as tools for the analysis of *Helicobacter* genome data. HelicoBase can be accessed at http://helicobacter.um.edu.my.

**Electronic supplementary material:**

The online version of this article (doi:10.1186/1471-2164-15-600) contains supplementary material, which is available to authorized users.

## Background

*Helicobacter* is a genus of Gram-negative bacteria possessing a characteristic spiral shape [1]. In the past, they were classified as members of the *Campylobacter* genus, but the *Helicobacter* genus has been recognized since 1989; currently with 29 known species (*H. acinonychis*, *H. anseris*, *H. aurati*, *H. bilis*, *H. bizzozeronii*, *H. brantae*, *H. canadensis*, *H. canis*, *H. cetorum*, *H. cholecystus*, *H. cinaedi*, *H. cynogastricus*, *H. felis*, *H. fennelliae*, *H. ganmani*, *H. hepaticus*, *H. mesocricetorum*, *H. marmotae*, *H. muridarum*, *H. mustelae*, *H. pametensis*, *H. pullorum*, *H. pylori*, *H. rappini*, *H. rodentium*, *H. salomonis*, *H. trogontum*, *H. typhlonius*, *H. winghamensis*) [2, 3]. *Helicobacter* species have been found living in the lining of the upper gastrointestinal tract, as well as the liver of some birds and mammals [4]. *Helicobacter* bacteria can be isolated from feces, saliva and dental plaque of some infected people which is consistent with known transmission routes [5–7]. The most widely known and well-studied species of the genus is *H. pylori*, a human pathogen which infects up to half of the human population [8]. In general, most patients with *H. pylori* infections do not have specific clinical symptoms or signs [9]. However, acute infection may appear as acute gastritis which may further develop into chronic gastritis if no treatment is given [10]. *H. pylori* is also strongly associated with peptic ulcers, duodenitis and stomach cancer [11]. *H. pylori* is a genetically diverse species that has co-evolved with the human race since their migration out of Africa 60,000 years ago [12], and subsequent geographic separation plus founder effects have resulted in distinct populations of bacterial strains that are specific for various geographical regions. In all, 7 populations and 3 subpopulations have been described: hpEurope (isolated from Europe, the Middle East, India and Iran), hpNEAfrica (isolated in Northeast Africa), hpAfrica1 (isolated from countries in Western Africa and South Africa), hpAfrica2 (so far only isolated from South Africa), hpAsia2 (isolated from Northern India and among isolates from Bangladesh, Thailand and Malaysia), hpSahul (from Australian Aboriginals and Papua New Guineans) and hpEastAsia with the subpopulations hspEAsia (from East Asians), hspMaori (from Taiwanese Aboriginals, Melanesians and Polynesians) and hspAmerind (Native Americans) [13–17].

With advances in next-generation sequencing technologies, many genomes of *Helicobacter* isolates have been sequenced by many laboratories [18–22]. The availability of these genome sequences from different sources has made it possible to get a deeper understanding of *Helicobacter* at the genomic level, for example through genome-wide comparative analyses. Such comparative analysis will have a profound impact on understanding the evolution, biology, diversity, evolution and pathogenicity of the *Helicobacter* spp. which may be useful in successfully managing *Helicobacter*-caused diseases.

Many specialized genomic databases or resources have been developed and published for well-studied human pathogens such as *Pseudomonas* Genome database [23, 24], *Burkholderia* Genome Database [25], Cyanobacteria Gene Annotation Database (CYORF) [26] and *Mycobacterium abscessus* Genome and Annotation Database (MabsBase) [27]. But no such specialized genomic database is available for *Helicobacter* spp. despite the wealth of available data. Microbial Genome Database for Comparative Analysis (MBGD) [28] and the Integrated Microbial Genomes (IMG) system [29] do provide a wide array of microbial genomes including some *Helicobacter* strains for comparative genomics, but lack the virulence factor perspective for comparative pathogenomics. Another concern regarding most of the existing biological databases is their lack of user-friendly web interfaces, for example, allowing real-time and fast querying and browsing of genomic data.

To facilitate *Helicobacter* research, we have developed a specialized *Helicobacter* resource and analysis platform for the storage of the rapidly increasing genomic data of *Helicobacter*, which presents the data in a useful manner that is easy to access, and enables the analysis of these genomic data, particularly in the field of comparative genomics. The aims of HelicoBase are to provide a comprehensive set of genomic data and a set of useful analysis tools with diverse functionality for data analysis. For instance, HelicoBase is powered by two newly designed tools: PGC for pairwise genome comparison and PathoProT for comparative pathogenomics analysis. The AJAX-based real-time search feature and JBrowse [30] have also been integrated into HelicoBase to allow rapid and seamless searching and browsing of the *Helicobacter* genomic data and annotations. Here we provide an overview and describe some key features of HelicoBase.

## Construction and content

**Figure 1:**
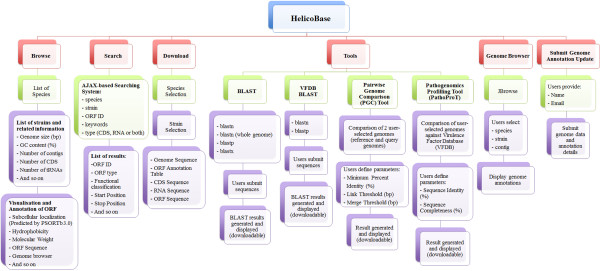
**Overview of HelicoBase.** There are 5 main functionalities accessible from the navigation bar on the top of the webpages: Browse, Search, Download, Tools, and Genome Browser. There are four analysis tools incorporated in HelicoBase: standard BLAST, VFDB BLAST, Pairwise Genome Comparison (PGC), and Pathogenomics Profilling Tool (PathoProT).

HelicoBase has much useful functionality as shown in Figure 1. In the homepage, users can view the latest news & conferences, blogs & information, and the most recent papers related to *Helicobacter* spp. that we manually compiled from different sources. By clicking on the ‘Browse’ hyperlink on the homepage, users can browse general information on different *Helicobacter* species (Table 1), where each species is linked, e.g. through the “View Strains” button, to a table showing all available strains (either draft or complete genome) and associated strain information like genome size, GC content, number of contigs, CDSs, number of tRNAs and number of rRNAs. Each species has a ‘Details’ button which directs users to the list of all RAST-predicted Open Reading Frames (ORFs). Useful ORF information is provided including ORF ID, ORF type, functional classification, contig ID, start position and stop position. If users want more information about a specific ORF, they can click on the “Detail” button provided for the ORF. This will direct users to an ORF details page with information like subcellular localization, hydrophobicity, molecular weight, and amino acid and nucleotide sequences of the ORF of interest. JBrowse is integrated into the ORF details page, allowing users to visualize and browse around the genomic location of the ORF. All these annotation details and sequence data for the selected ORF can be downloaded in the same page as CSV and FASTA files, respectively. Furthermore, users can also download the whole-genome annotations and sequences through the provided ‘Download’ page.

**Table 1:** **List of** ***Helicobacter*** **species and genomes in HelicoBase**

#	Species	Number of draft genomes	Number of complete genomes
1	*H. acinonychis*	0	1
2	*H. bilis*	1	0
3	*H. bizzozeronii*	1	1
4	*H. canadensis*	2	0
5	*H. cetorum*	0	2
6	*H. cinaedi*	1	1
7	*H. felis*	0	1
8	*H. hepaticus*	0	1
9	*H. mustelae*	0	1
10	*H. pullorum*	1	0
11	*H. pylori*	106	43
12	*H. suis*	2	0
13	*H. winghamensis*	1	0

HelicoBase currently accumulates a total of 166 genome sequences from 13 *Helicobacter* species, which were downloaded and compiled from the National Center for Biotechnology Information (NCBI) [31, 32]. To have consistent annotations for comparative analyses, we re-annotate all genomes with the Rapid Annotation using Subsystem Technology (RAST) pipeline [33]. RAST has been successfully tested in annotating both complete and draft genomes of archaea and bacteria in the recent review by Liu et al. [34]. Using this well-established pipeline, functional elements like protein-encoding genes, rRNAs, tRNAs and pseudogenes can be predicted in each *Helicobacter* genome. All genome annotations were stored in our MySQL database. Currently HelicoBase has stored 280,550 coding sequences (CDSs), 6,683 rRNAs and 5,965 tRNA genes predicted in all 166 genomes of the 13 *Helicobacter* species. Among annotations generated by RAST include ORF type, functional classification, chromosomal position, nucleotide length, amino acid length and strand. Other annotations like subcellular localisation, hydrophobicity and molecular weight of the RAST-predicted proteins are also provided. For subcellular localization prediction, we used PSORTb version 3.0, a well-established software to determine the subcellular localization of putative proteins for prokaryotes [35]. In HelicoBase, the 280,550 RAST-predicted CDSs were categorised by PSORTb into 5 different categories such as cytoplasmic, cytoplasmic membrane, extracellular, outer membrane and periplasmic (Additional file 1: Figure S1).

### Real-time data searching feature

**Figure 2:**
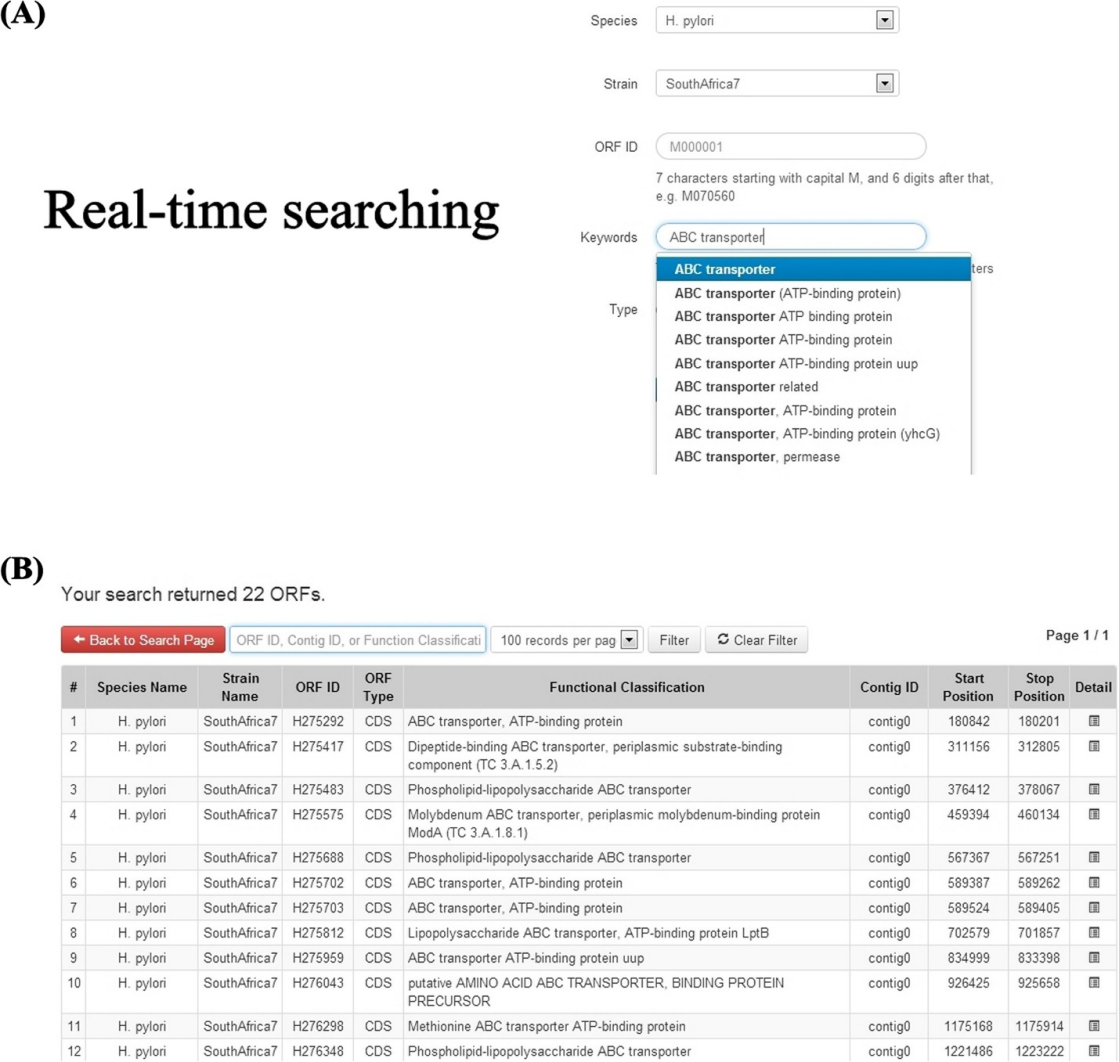
**Real-time search feature. (A)** Example of Real-time searching with *H. pylori* strain SouthAfrica7 with “ABC transporter” as keyword. A list of matches with the typed keyword was retrieved from HelicoBase in a real-time manner. **(B)** Example of search output.

With advances in next-generation sequencing technologies and bioinformatics, it is anticipated that the data in HelicoBase will considerably increase as more genomes are sequenced in the future. Therefore, a user-friendly interface allowing users to rapidly search a massive amount of genomic data is vital. To give the *Helicobacter* research community a user-friendly and seamless search experience, we have implemented a powerful real-time AJAX-based search system in the “Search” page on the homepage. Users can search for an ORF by using different parameters including species name, strain, ORF ID, keywords of functional classification and type of sequence (Figure 2). Furthermore, when users type in the search keywords, the system will rapidly retrieve the matches from HelicoBase in a real-time manner. This will help users to get the right keywords and will speed up their searching, which is vital in searching a huge database.

## Utility

### Pairwise genome comparison (PGC) tool: information aesthetic for comparative genomics

HelicoBase is not just designed as a genomic data repository, but also aims to be an analysis platform, particularly to facilitate comparative analysis of multiple *Helicobacter* genomes. The PGC tool is a newly designed in-house comparative analysis tool allowing users to compare two selected *Helicobacter* genomes and display the results in a circular layout on the fly. Through the provided web interface of PGC, users can choose two genomes of interest in HelicoBase for comparison. Alternatively, users can use an online custom web form to upload their own *Helicobacter* genome sequence for comparison with a *Helicobacter* genome in HelicoBase.

**Figure 3:**
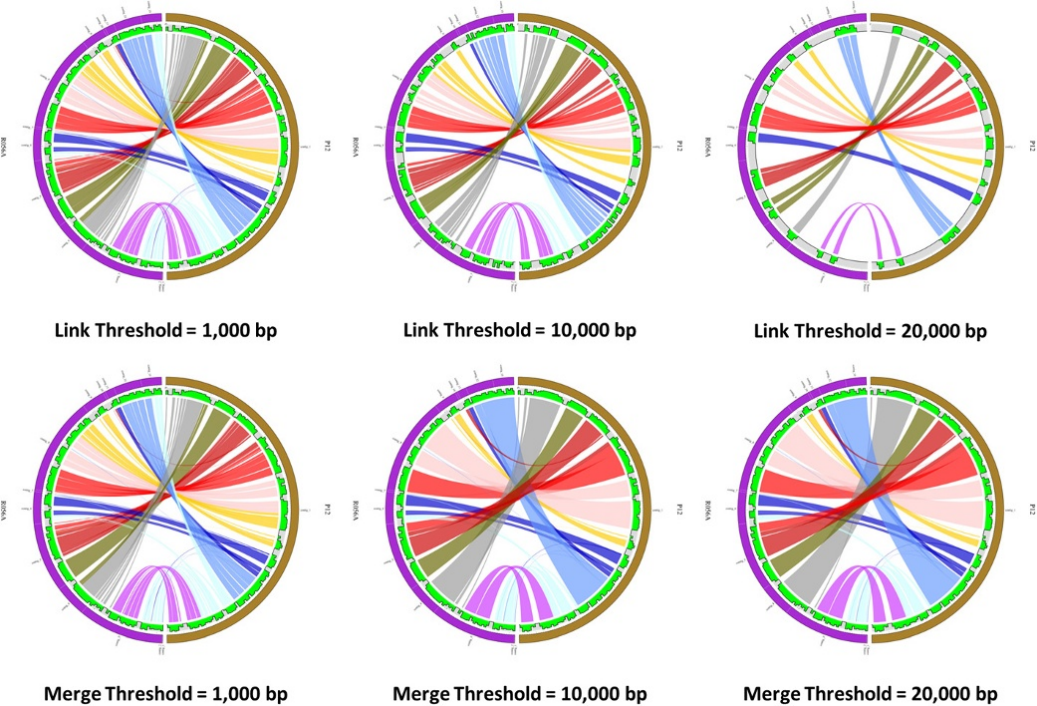
**Output of different cut-offs for the Link Threshold (LT) and Merge Threshold (MT) when comparing** ***H. pylori*** **R056A and** ***H. pylori*** **P12.** Different user-defined cut-offs affect the output display of the two aligned genomes. The top three plots were generated at genome identity of 95% and MT of 0 bp, but at different LT cut-offs. The three plots at the bottom were generated at genome identity of 95% and LT of 1,000 bp, but at different MT cut-offs. Each half circle (either left or right) represents each separate genome/assembly. The coloured links show the homologous regions in the two selected genomes. The green track is the alignment histogram; each 10 Kbp window in the diagram is represented by a histogram bar and the height of each bar illustrates the total number of bases of the opposite genome aligned to this 10 Kbp window region. The upper border of the grey area delineates 10 Kbp height. If the height is higher than the 10 Kbp, it may indicate non-specific alignment or windows containing repetitive regions. A trough may indicate an unmapped region which could be an insertion e.g. prophage insertion. We can clearly observe how different user-defined thresholds affect the display of the two aligned genomes.

Three main parameters are provided: the minimum percent identity (%), merge threshold (bp) and link threshold (bp). By default, the thresholds of the PGC tool are set to be 95% minimum percent identity and 1,000 bp link threshold. But users may change the parameter freely to get different comparative results. The influences of different parameters on the display of the aligned genomes with Circos are shown in Figure 3. The details of how the merge threshold works is shown in Additional file 2: Figure S2A.

**Figure 4:**
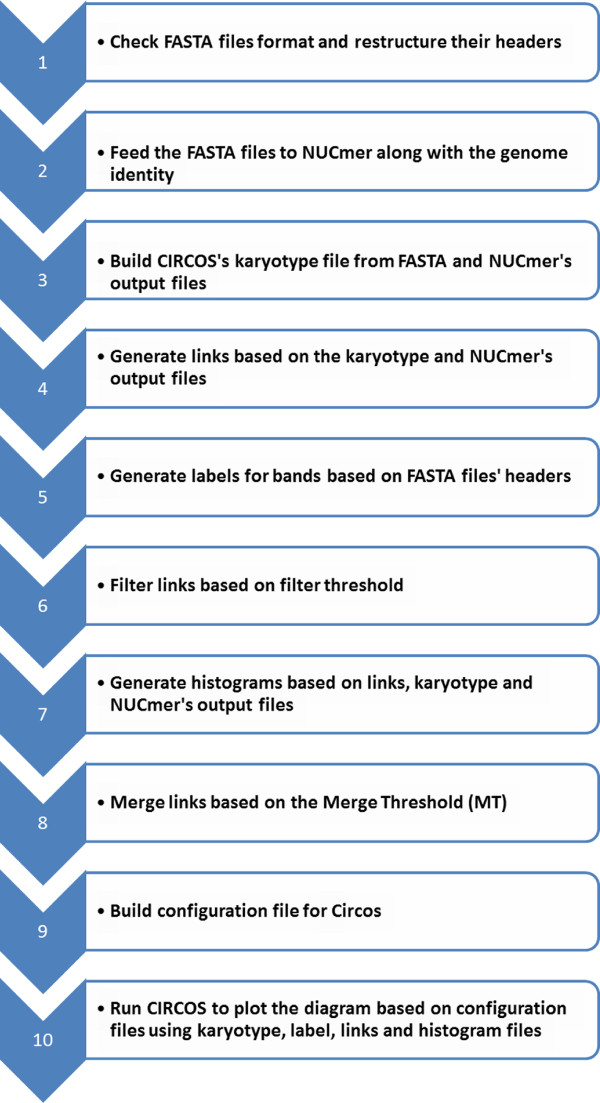
**A flow chart that briefly illustrates the processes involved in PGC Pipeline after a job is submitted to our server.**

Once a user submits their job to our server, PGC will align both genome sequences using NUCmer, from the MUMmer package [36]. Our pipeline will process the output files generated by NUCmer and generate a few input files (configuration file, karyotype file and so on) to be used to generate a circular graphic plot using Circos, which is a powerful tool to display the relationship between the two aligned genomes [37]. The circular representation of the two aligned genomes provides a clear view of the similarities and differences (e.g. indels and rearrangements) in genome structure of the selected *Helicobacter* strains. The detailed workflow on how PGC works after users submit their jobs to our server is shown in Figure 4.

**Figure 5:**
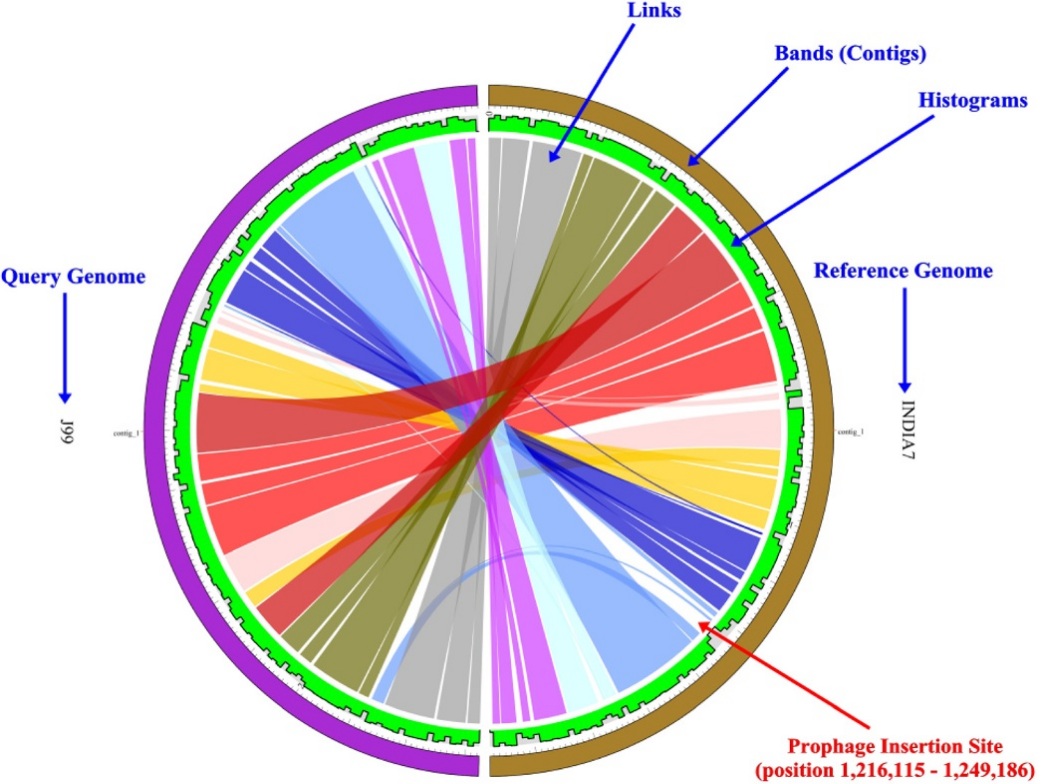
**Analysis of two closely related** ***Helicobacter*** **strains using PGC tool.** Genome comparison are performed between *H. pylori* J99 and *H. pylori* India7. The “flat” pattern in the histogram track indicates the two genomes are generally conserved/similar, whereas the gaps may indicate unaligned genomic regions e.g. indels. A large indel was predicted as prophage sequence.

As a case study, we compared the genomes of two closely related *Helicobacter* strains, *H. pylori* J99 and *H. pylori* India7 using PGC (Figure 5). In general, both genomes are conserved. However, we still can observe differences e.g. indels between the genomes. Further analysis on one of the large indels in the genome of *H. pylori* India7 revealed a putative intact prophage as predicted by PHAge Search Tool (PHAST) [38]. The observation of the intact prophage suggests that it might be recently inserted into the genome of *H. pylori* India7 through Horizontal Gene Transfer (HGT). The introduction of the intact prophage in the genome of *H. pylori* India7 strain has probably conferred pathogenicity to the bacterial host and may represent an adaptation to different environments. This example demonstrates the usefullness of PGC for viewing and interpreting the genetic differences between two genomes.

Although a similar tool, called Circoletto [27] is available, PGC has some advantages over this online tool. Firstly, while Circoletto aligns sequences using BLAST (local alignment) the alignment algorithm used in PGC is based on the NUCmer (global alignment) package in MUMmer, which is suitable for large-scale and rapid genome alignment. Secondly, PGC provides many useful options for users. For instance, a user can adjust settings such as minimum percent genome identity (%), merging of links/ribbons according to MT, and the removal of links according to the user-defined LT through the provided online form. Thirdly, in the circular layout generated by PGC, a histogram track showing the percentage of mapped regions along the genome is provided. This track is very useful and helps users to identify putative indels and repetitive regions in the *Helicobacter* genomes. Additional file 2: Figure S2B shows how the data in the histogram track is calculated. Besides the Circoletto, RCircos is another tool with a similar function to PGC, which was developed by Zhang et al. [39]. RCircos was developed using R packages that come with R base installation. The package supports Circos 2D data track plots such as scatter, line, histogram, heat map, tile, connectors, links, and text labels [40]. However, unlike PGC which has a user-friendly interface and is easy to use without knowledge in programming, users need to have knowledge in the R programming language in order to run the RCircos and no user-friendly interface is provided.

### A newly designed pathogenomics profiling tool (PathoProT) for comparative pathogenomics analysis

Virulence factors are molecules present in bacteria, which are responsible for causing disease in the host or converting non-pathogenic bacteria into pathogens [41, 42]. The availability of sequenced genomes of different *Helicobacter* species makes the comparative analyses of virulence factors in the *Helicobacter* pathogen genomes feasible and may provide new insights into pathogen evolution and the diverse virulence strategies employed. Understanding the pathogenic mechanisms of these pathogens would aid in the treatment and prevention of *Helicobacter*-caused diseases.

**Figure 6:**
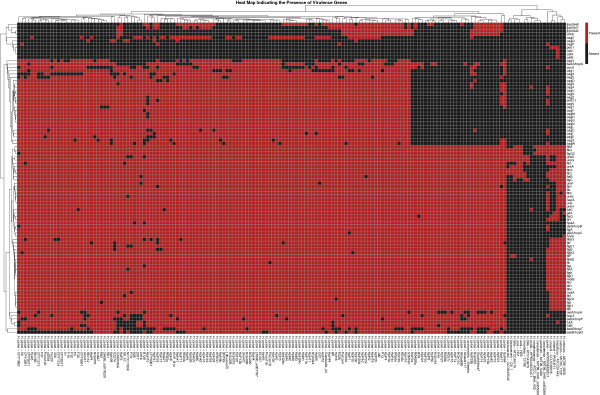
**A heat map generated by PathoProT.** All strains in HelicoBase were used to generate this heat map.

**Figure 7:**
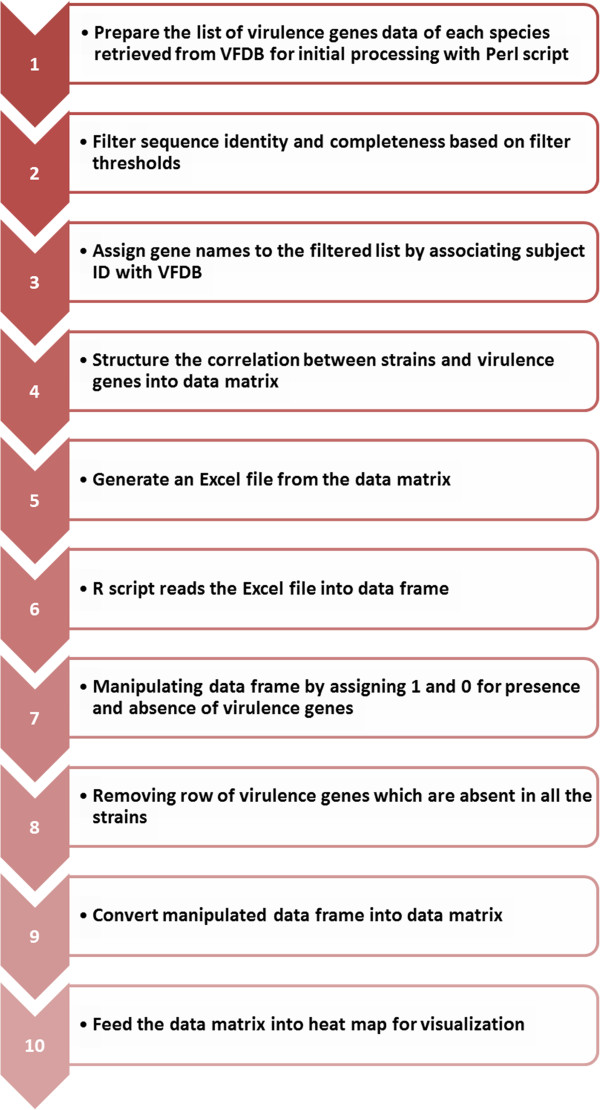
**Flow chart that briefly illustrates the processes involved in PathoProT Pipeline.**

To identify virulence genes and facilitate the comparative pathogenomics analysis of multiple bacterial species/strains, we have developed a unique Pathogenomics Profiling Tool (PathoProT). PathoProT predicts virulence genes based on sequence similarity by BLASTing all RAST-predicted protein sequences in user-selected strains against the VFDB [43–45]. A gene will be defined as a virulence gene if it has a BLAST hit that meets defined cut-offs e.g. 50% sequence identity and 50% sequence completeness set by the users. Once the putative virulence genes are identified in each user-defined strain, PathoProT will cluster (agglomerative hierarchical cluster analysis) the virulence genes and strains based on their virulence gene profiles and visualize them as a heat map with dendrograms. Through the heat map, users can examine the similarities and differences of the virulence gene profiles between different groups of strains e.g. non-pathogenic versus pathogenic strains (Figure 6). The detailed steps involved in our PathoProT pipeline after the BLAST searches are completed are shown in Figure 7. For more details on the usage of PathoProT tool, we included a “Help” page in the PathoProT tool, aimed to provide definitions and support to users.

Figure 6, gives a bird eye’s view of the virulence genes that are present and widely distributed across all *Helicobacter* species. In general, it is clearly shows that *H. pylori* strains have more virulence genes compared to other species, which may explain their high virulence [46, 47].

Interestingly, *H. hepaticus* ATCC51449 harbours three unique virulence genes, which are *cdtA*, *cdtB* and *cdtC*. The cytolethal distending toxins (CDTs) constitute the most recently discovered family of bacterial protein toxins. CDTs are unique among bacterial toxins as they have the ability to induce DNA double strand breaks in both proliferating and non-proliferating cells, thereby causing irreversible cell cycle arrest or death of the target cells [48]. It has been shown that CDTs encoded by the three genes, *cdtA, cdtB*, and *cdtC* are required for cytotoxicity [49]. When cdtA, *cdtB*, and *cdtC* are present together, the CDTs interact with one another to form an active tripartite holotoxin. The presence of these three genes in *H. hepaticus* ATCC51449 is supported by a recent study by Vincent et al., who identified these virulence genes in *H. hepaticus* species [50].

In summary, we have demonstrated that PathoProT can be used to identify virulence genes in *Helicobacter* strains by sequence homology. Moreover, comparative pathogenomics analysis can be easily performed to compare strains/groups of strains e.g. non-pathogenic strains versus pathogenic strains, which can give better insights into the biology, evolution and virulence of the *Helicobacter* strains of interest. In other words, PathoProT can be used to answer interesting biological questions including what are the conserved virulence genes in a group of *Helicobacter* strains and enables strain-specific/group-specific virulence genes to be easily viewed in the generated heat map.

### Other tools

BLAST is included in HelicoBase to allow for easy similarity searching for sequences of interest [51]. The built-in BLAST in HelicoBase provides two main functions: (1) standard BLAST which will search the provided query sequence against genome or ORF sequences (either nucleotide or protein) in HelicoBase; (2) VFDB BLAST which searches the provided query sequence against the Virulence Factor Database (VFDB) [43, 44, 52]. Users can use VFDB BLAST if they want to determine whether their sequence of interest is a virulence gene based on sequence homology.

**Figure 8:**
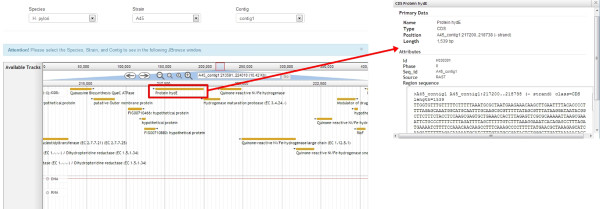
**A sample output of JBrowse in HelicoBase.** A genomic region of the contig 1 of *H. pylori* A45 was visualised in JBrowse. Clicking on gene encoding for protein hydE displays a pop-up window with the useful information associated with the gene.

JBrowse is another tool that we have integrated into HelicoBase to give users a seamless browsing experience [30] (Figure 8). This next generation AJAX-based genome browser built with JavaScript and HTML5 enables the user to explore the genome of interest with unparalleled speed and scales easily to multi-gigabase genomes and deep-coverage sequencing [45]. JBrowse preserves the user’s sense of location by avoiding discontinuous transitions, offering smooth and fast animated panning, zooming, navigation and track selection. With the advances in next-generation sequencing technologies and bioinformatics tools, we anticipate that many more *Helicobacter* genomes will be sequenced and annotated. Therefore, a user-friendly JBrowse that allows rapid and seamless browsing of high volumes of genomic data will be a major advantage.

### HelicoBase development and implementation

HelicoBase rests on MySQL version 14.12 (http://www.mysql.com) and was hosted using Ubuntu Lucid 10.04 Web server application (http://www.ubuntu.com). Development used a combination of PHP 5.3 and Perl 5 languages, with Codelgniter 2.1.3 framework for web tier and Twitter Bootstrap front-end framework for the presentation layer.

The web server architecture was designed to be scalable and combined with a flexible PHP coding interface enables users with different devices to connect to HelicoBase in a fast and readable manner. For tools such as BLAST, VFDB BLAST, PathoProT, and PGC users can submit their analysis jobs through the provided interfaces and these jobs will be submitted to the cluster server in a firewall-enabled secure process. The job scheduler in turn makes it possible for the application server to process the submitted jobs in a fair and parallel manner with fast processing speed. Our cluster computer (5 nodes, 12 CPUs for each node and 625GB of RAM in total) prompts the database server to run in a fast fibre-optic internal network to retrieve necessary information needed to process the submitted jobs. Meanwhile, our MySQL data structures were formed in a manner that supports fast and localized searching which makes the user-website interactions fast and also user-friendly. To construct HelicoBase, we used the several software components: RAST [33], BLAST [51], MUMmer [53], PSORTb [54], Circos [37] and JBrowse [30].

## Discussion and conclusion

With advances in high-throughput sequencing technologies, it is imperative that the abundant data generated can be easily accessible for analysis. With HelicoBase we aim to provide a one-stop resource platform that will make it easy to access and analyse whole-genome genomic data and information for *Helicobacter* spp. through an organised and user-friendly interface. PGC and PathoProT are some of the bioinformatics tools for comparative analysis implemented in HelicoBase which allow researchers to conveniently assimilate and explore the data in an intuitive manner.

HelicoBase will be updated from time to time as more genome sequences of *Helicobacter* spp. become available. To accelerate the development of HelicoBase, we encourage researchers to email us at girg@um.edu.my if they would like to share their annotations and related data with us. Suggestions on improving HelicoBase are most welcome.

## Availability and requirements

HelicoBase is available online at http://helicobacter.um.edu.my. All sequences and annotations described in this paper can be downloaded from that site.

## Electronic supplementary material

Additional file 1: Figure S1: Classification of CDS Subcellular Localization in HelicoBase. Protein-coding genes with ambiguous and low subcellular scores were classified into unknown category. (PDF 68 KB)

Additional file 2: Figure S2: (A) A diagram showing how the merge threshold works with the merging process by PGC tool. Merge Threshold provides users with the ability to ignore minimal spaces between adjacent links. Adjacent links are those which are adjacent in their position in both of the genomes. (B) A diagram showing how the data in histogram track was calculated based on different scenarios. Basically histogram bars delineate the total length of links (bp) that are mapped to a particular window. The window denotes 10 kbp slices of genomes. Note that having bar with the height equal to borderline does not necessarily mean that the whole window is covered with links. All this information is available on the ‘Help’ icon provided in PGC tool. (PDF 4 MB)
