# Supplementary figures and images for: HelicoBase: a Helicobacter genomic resource and analysis platform
Source: BMC Genomics. 2014 Jul 16;15(1):600. doi: 10.1186/1471-2164-15-600 (PMC4108788; doi:10.1186/1471-2164-15-600)

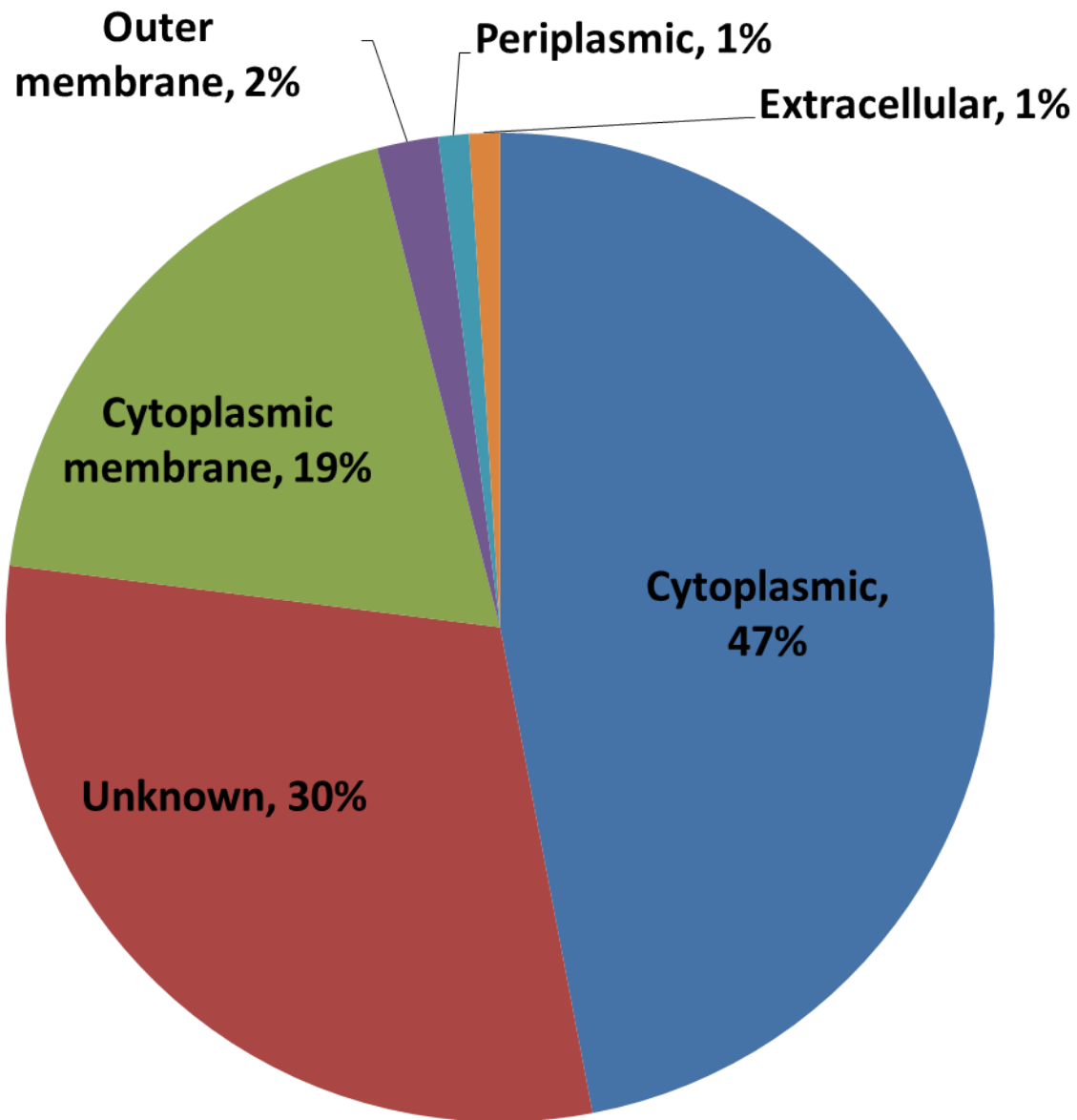

**Protein Subcellular Localization**

Supplement: Supplementary file 1 — Additional file 1: Figure S1: Classification of CDS Subcellular Localization in HelicoBase. Protein-coding genes with ambiguous and low subcellular scores were classified into unknown category. (PDF 68 KB) [file 12864_2014_6269_MOESM1_ESM.pdf]

(A)

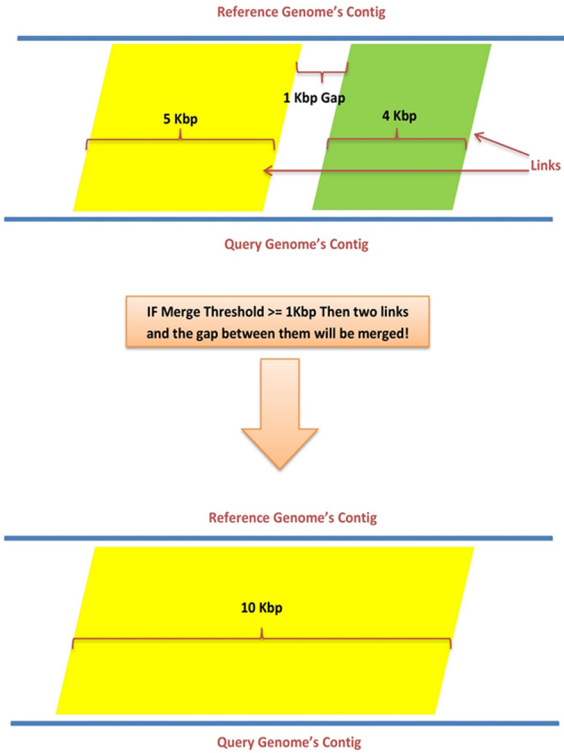

(B)

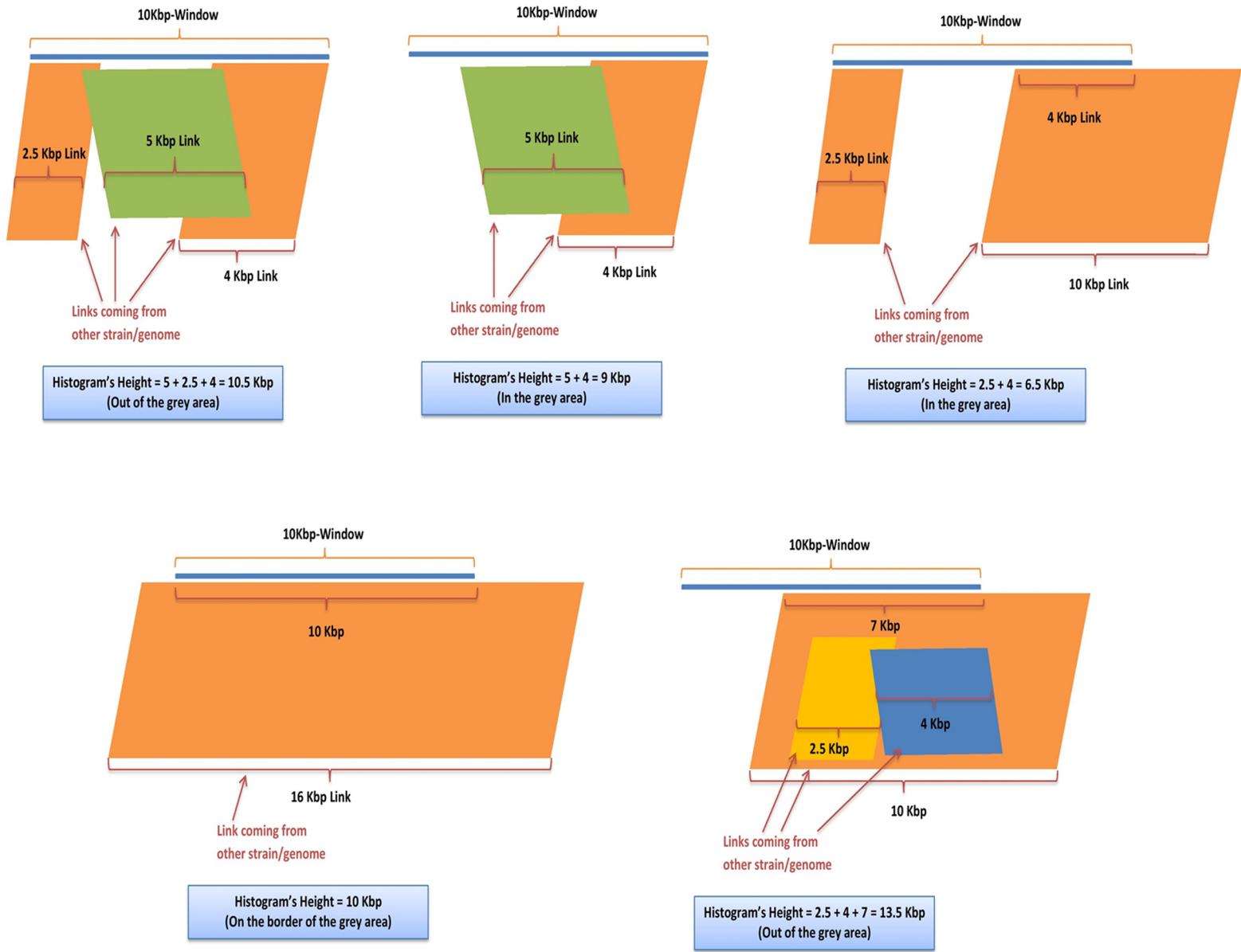

Supplement: Supplementary file 2 — Additional file 2: Figure S2: (A) A diagram showing how the merge threshold works with the merging process by PGC tool. Merge Threshold provides users with the ability to ignore minimal spaces between adjacent links. Adjacent links are those which are adjacent in their position in both of the genomes. (B) A diagram showing how the data in histogram track was calculated based on different scenarios. Basically histogram bars delineate the total length of links (bp) that are mapped to a particular window. The window denotes 10 kbp slices of genomes. Note that having bar with the height equal to borderline does not necessarily mean that the whole window is covered with links. All this information is available on the ‘Help’ icon provided in PGC tool. (PDF 4 MB) [file 12864_2014_6269_MOESM2_ESM.pdf]
